# Inadvertent Skipping of Steroids in Septic Shock Leads to a Diagnosis of Adult Onset Still’s Disease

**DOI:** 10.7759/cureus.978

**Published:** 2017-01-14

**Authors:** Vinoth K Sethuraman, Kavitha Balasubramanian, Stalin Viswanathan, Rajeswari Aghoram

**Affiliations:** 1 Department of General Medicine, Indira Gandhi Medical College & Research Institute, Pondicherry, India

**Keywords:** still’s disease, shock, pneumonia, hyperferritinemia, elderly

## Abstract

Adult onset Still’s disease is uncommon in middle-aged and elderly individuals and can rarely present with shock; shock is usually associated with disseminated intravascular coagulation, multiorgan dysfunction syndrome or acute respiratory distress syndrome. We report a post-menopausal woman with arthritis, fever, pneumonitis and hypotension which was managed as septic shock. Steroids were inadvertently missed during the second day of hospitalization in the intensive care unit. Persistence of hypotension on inotropes, with normal renal, hepatic and neurological function and recurrence of fever when steroids were skipped, led to suspicion of an inflammatory disorder. A diagnosis of Still’s disease may be entertained in postmenopausal women with polyarthritis, rash, and fever with leukocytosis. Sepsis is mimicked, and multiple antibiotics use is common before the diagnosis of such an entity is made. Shock is rare in adult onset Still’s disease and is not necessarily associated with disseminated intravascular coagulation, acute respiratory distress syndrome, or multiorgan dysfunction.

## Introduction

Adult-onset Still’s disease (AOSD) is a rare systemic inflammatory disorder of unknown etiology, first described in children by Sir George Frederic Still in 1897 [1]. Still’s disease usually presents with high spiking fever, arthralgia or arthritis, sore throat, transient maculopapular rash, lymphadenopathy, hepatosplenomegaly, and serositis. The disease mainly affects young adults, and has a bimodal age distribution—15–25 and 36–46 years of age [1]. AOSD may occasionally present in older individuals [2]. We describe the case of a middle-aged female with pneumonitis and septic shock, in whom skipping steroids inadvertently in the intensive care unit (ICU) led to consideration of an inflammatory disorder and a diagnosis of AOSD. Informed consent was obtained from the patient for this study.

## Case presentation

**Figure 1:**
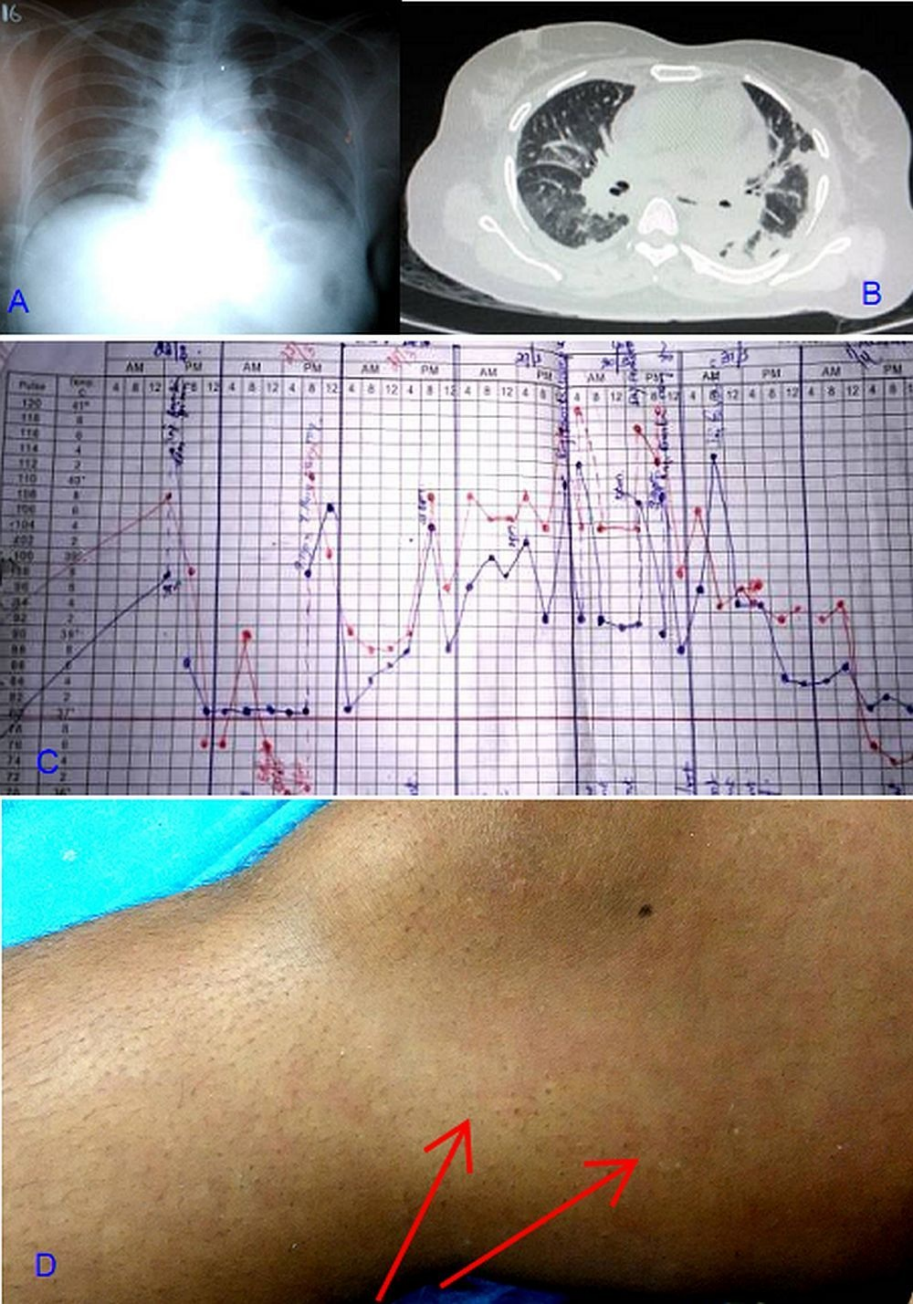
The patient's imagings, fever chart, and rash a. Chest radiograph showed cardiomegaly and left costophrenic angle blunting. b. Chest computed tomography revealed bilateral mild pleural effusion with consolidation along the medial basal segment of the left lower lobe and medial segment of the right middle lobe. c. Temperature chart shows fever crashing on day 2 (first day in ICU) and high spiking temperature for the next four days. d. Right thigh shows salmon-colored rash, which appeared on day 4.

A 50-year-old postmenopausal woman with no comorbid illnesses was admitted to another hospital with polyarthralgia of two weeks duration and had been initiated on analgesics for probable seronegative rheumatoid arthritis. Two days later, she presented to our emergency department (ED) with high-grade fever with chills, cough, abdominal pain, and increasing arthralgias of both knees, ankles, wrists and elbow joints. On admission, she was febrile (102 °F), hypotensive (80/50 mm Hg), tachypneic (34 breaths/min), and had tenderness of bilateral knee, ankle and wrist joints. Her systemic examination showed bilateral basal crackles and epigastric tenderness. The patient was diagnosed to have bilateral pneumonitis, sepsis with septic shock and probable drug-induced gastritis. Pending cultures and serological investigations, she was empirically administered ceftriaxone, azithromycin, hydrocortisone (50 mg q6h, received two doses) and noradrenaline and shifted to the ICU. Investigations (Table 1) in the ED revealed neutrophilic leukocytosis, ESR 85 mm/hr, bilateral perihilar opacities with left pleural effusion (Figure 1a) and respiratory alkalosis. In the ICU, intravenous fluids and noradrenaline were continued. Intravenous hydrocortisone was inadvertently missed while repeating the physician’s orders. The patient remained afebrile for 12 hours during the first day in ICU (Figure 1c), but the hypotension did not improve. With the recurrence of fever on the third day, piperacillin-tazobactam + linezolid was substituted for ceftriaxone. Pantoprazole and paracetamol (500 mg q6h) were also administered. In view of normal hepato-renal function and neurological status and normal echocardiography on the third day of shock, septic shock was considered less likely and the decision to restart steroids deferred. The patient remained hypotensive and febrile until the fourth day of admission when she also developed a sore throat, bilateral knee joint and ankle joint swelling with effusion, and a salmon-colored blanching macular rash over the trunk and extremities (Figure 1d). The temperature chart showed daily high spiking fevers touching 40 °C, interrupted by an afebrile period during the first day in ICU. With these new complaints and fever chart review, steroids were reinitiated on day 5, pending further investigations (antinuclear antibodies (ANA), anti-double stranded DNA (dsDNA) and ferritin).

**Table 1:** Investigations of the patient during her hospital stay WBC-white blood cell; ESR-erythrocyte sedimentation rate; HBsAg- Hepatitis B surface antigen; HIV-human immunodeficiency virus; ds-DNA-double stranded DNA

Day	Day 1	Day 3	Day 5	Day 9	Day 14		
WBC x10^9^/L	34.4	36	30.9	22.3	19.7		
Neutrophils %	88	89	88	84	75		
Platelets x10^9^/L	364	376	330	730	751		
ESR	80	125			45		
Blood culture	*Sterile *	Lepto IgM	*Negative*	HBsAg	*Negative*	ds-DNA	*Negative*
Urine culture	*Sterile*	Scrub IgM	*Negative*	HIV	*Negative*	RF	*Negative*
Malaria	*Negative *	Widal	*Negative*	AntiHCV	*Negative*	ASO	*Negative*

Investigations (Table 1) revealed persistent neutrophilic leukocytosis with toxic granules and positive C-reactive protein (CRP) (1:4 dilution). Serum ferritin was grossly elevated (12563.80 mg/ml; range 10–291). Antinuclear antibodies (ANA) was moderately positive. Procalcitonin was unavailable in our institution. Ultrasound of the knees showed bilateral mild to moderate knee joint effusion with mild synovial thickening. Chest and abdominal computed tomography revealed bilateral mild pleural effusion with consolidation in left lower lobe and right middle lobe, and mild hepatomegaly (Figure 1b). Based on her history, clinical examination, and laboratory investigations, a diagnosis of AOSD with pneumonitis, pleuritis, and shock were made using Yamaguchi criteria (4 major +2 minor). The next day she became afebrile and normotensive. The joint swelling and pain began resolving and she became asymptomatic after five days. She was discharged from the hospital with tapering dose of oral prednisolone and methotrexate on the 15th day of admission. She made two follow-up visits after leaving the hospital and did not have further symptoms.

## Discussion

The prevalence of AOSD is estimated to be 1/100,000 people. AOSD has rarely been reported in the elderly [2]. The most common clinical features of AOSD are arthralgia (98–100%), fever (83–100%), myalgia (84–90%), rash (87–90%) and sore throat (50–92%) [3]. The joints involved include the knees, wrists, ankles, elbows, metacarpophalangeal, metatarsophalangeal and distal interphalangeal joints. The disease pattern of patients was previously divided into three distinct types: monocyclic or self-limiting pattern, polycyclic or intermittent pattern, and chronic articular pattern [3]. Recent data suggests that there are two types, based on their cytokine profile, clinical features, and response to treatment: systemic AOSD and articular AOSD. Hyperferritinemia, high fever, and serositis are more suggestive of systemic AOSD as in our patient. These patients respond well to first-line corticosteroid therapy. Activation of macrophages and neutrophils, reduced cytotoxicity of natural killer (NK) cells, elevated cytokines such as interleukin (IL)-18 and 1β, tumor necrosis factor (TNF)α and chemokines (CXCL8, a neutrophil inducer) have been shown in AOSD. IL-18 induces TH1 cytokine production, while IL-1β primes NK cells to secrete TNFγ. TNF α levels do not correlate with disease activity. IL-1 antagonists and TNFα blockers have been used in refractory AOSD, suggesting a prominent role for autoimmunity. Dreaded complications such as reactive hemophagocytic lymphohistiocytosis are more common in systemic AOSD [3].

Less commonly, lymphadenopathy, hepatomegaly, splenomegaly, pericarditis, myocarditis, stroke, aseptic meningitis, cranial nerve palsies, glomerulonephritis and thrombotic thrombocytopenic purpura are observed [1]. About 50% of patients have abdominal pain. Transient pulmonary infiltrates and pleural effusion are the most common pulmonary manifestations [4]. Rarely, patients may develop acute respiratory distress syndrome (ARDS), cryptogenic organizing pneumonia and diffuse pulmonary hemorrhage (DAH) [5]. In a series by Detorakis, et al., pleural effusion, consolidation/atelectasis, ground glass opacities, interlobular septal thickening, nodules/micronodules, bronchiolar involvement and air trapping were seen [Detorakis E, Magkanas E, Matalliotaki P, et al. Thoracic involvement in adult-onset Still's disease (AOSD): spectrum of imaging findings in chest radiograph and computed tomography. Poster presented at ECR 2010/C-0999, European Society of Radiology, Electronic Presentation Online System]. In a study by Zeng, et al., 11.5% had interstitial pneumonia on CT; six of the 61 patients in this series died, and four died from pneumonia/respiratory failure [6]. Severe pulmonary involvement, including pleuritis and interstitial pneumonia in AOSD, was associated with a poor prognosis. Our patient had ground glassing and pneumonitis with pleural effusion, which recovered.

Shock is extremely uncommon and is usually a part of multisystem fulminant disease or acute respiratory distress syndrome (ARDS) or disseminated intravascular coagulation (DIC), where hydrocortisone may not suffice and methyl prednisolone or biologic agents may be necessary [7-8]. Hyperferritinemia is observed in septic shock, antiphospholipid antibody syndrome, AOSD, and macrophage activation syndrome (MAS) [9]. Titers >10,000 are seen in AOSD and MAS. Serum ferritin level is suggested as a prognostic marker of AOSD and is higher than the level found in other autoimmune and inflammatory disorders. Procalcitonin, a marker of sepsis is also increased in non-septic AOSD. Testing for ANA and rheumatoid factor are negative in a majority of patients with AOSD [1].

## Conclusions

A diagnosis of AOSD can be entertained in postmenopausal women with polyarthritis, rash, and fever with leucocytosis. AOSD mimics sepsis, and multiple antibiotics use is common before a diagnosis is made. Shock is rare in AOSD and is not necessarily associated with DIC, ARDS, or multiorgan dysfunction. Scrutiny of fever charts gives clues to both diagnostic approaches as well as therapeutic errors.
